# Centesimal Composition, Bioactive Compounds, Antioxidant and α-Glucosidase Inhibitory Activities of Commercial Edible Oyster Mushrooms at Different Maturity Stages in Northern Thailand

**DOI:** 10.3390/foods14203511

**Published:** 2025-10-15

**Authors:** Jaturong Kumla, Saisamorn Lumyong, Nakarin Suwannarach

**Affiliations:** 1 Office of Research Administration, Chiang Mai University, Chiang Mai 50200, Thailand; Jaturong_yai@hotmail.com; 2 Center of Excellence in Microbial Diversity and Sustainable Utilization, Chiang Mai University, Chiang Mai 50200, Thailand; saisamorn.l@cmu.ac.th; 3 Department of Biology, Faculty of Science, Chiang Mai University, Chiang Mai 50200, Thailand; 4 Academy of Science, The Royal Society of Thailand, Bangkok 10300, Thailand

**Keywords:** bioactive compounds, developmental stages, edible mushroom, centesimal content, phenolic compounds

## Abstract

Oyster mushrooms (*Pleurotus* spp.) are widely cultivated due to their high nutritional value and bioactive compounds with health-promoting properties. However, the fruiting body developmental stage significantly influences the centesimal composition and bioactive compound levels. This study examined the centesimal composition and bioactive properties of five commercial oyster mushroom species (*P. citrinopileatus*, *P. cornucopiae*, *P. djamor*, *P. ostreatus*, and *P. pulmonarius*) cultivated in northern Thailand at three maturation stages (young, middle, and mature). The centesimal composition; polysaccharide, ergothioneine, and phenolic compound contents; antioxidant activity; and α-glucosidase inhibitory activity were analyzed. The results showed that the centesimal composition and polysaccharide content increased as the mushrooms matured in all species. The middle stage consistently exhibited the highest levels of ergothioneine, total phenolics, and individual phenolic compounds in all five species. Twelve phenolic compounds were identified, with 4-hydroxybenzoic acid, trans-cinnamic acid, and trans-o-coumaric acid being predominant. All extracts exhibited antioxidant activity, according to the 2,2′-azino-bis(3-ethylbenzothiazoline-6-sulfonic acid) (ABTS), 2,2-diphenyl-1-picrylhydrazyl (DPPH), and ferric reducing antioxidant power (FRAP) assays, and α-glucosidase inhibitory activity, with the highest activity found at the middle stage. This is the first paper to report the ergothioneine content and *α*-glucosidase inhibitory activity in *P. cornucopiae* and *P. djamor*. These findings demonstrate that harvest timing can be optimized to maximize either the nutritional content (mature stage) or bioactive compound content for functional food applications (middle stage), offering a better understanding of the developmental phases at which mushrooms have the greatest health and technological potential. Furthermore, this knowledge provides practical guidance for growers seeking to target specific markets for high nutritional value foods based on consumer demand and for functional food developers aiming to maximize health-promoting properties.

## 1. Introduction

Edible mushrooms represent a significant source of nutrients and bioactive compounds and are recognized for their unique taste profiles and health-promoting qualities [1,2,3]. They are rich in proteins, fiber, vitamins, minerals, and a variety of bioactive compounds [2,4,5]. Mushroom cultivation is a globally significant agricultural sector that has experienced substantial growth over the past decade. In 2023, global production of mushrooms and truffles reached 50 million tons, with an estimated market value of USD 46.8 billion [6]. Asia overwhelmingly dominates this production (48.1 million tons), with China being the leading producer. Within Southeast Asia, Vietnam is the largest producer, followed by Indonesia and Thailand [6]. Most cultivated edible mushrooms are saprophytic fungi (decomposers) that are capable of breaking down complex organic matter, particularly lignocellulosic materials, into accessible nutrients that support their growth and development [7,8]. As a result, mushroom cultivation is closely linked to the sustainable recycling of vast quantities of agricultural and agro-industrial residues, offering an environmentally friendly solution for managing biomass that would otherwise contribute to pollution [7,9]. Over 50 edible mushroom species are grown for commercial purposes globally, with the majority of cultivation focused on the genera *Agaricus*, *Agrocybe*, *Auricularia*, *Flammulina*, *Ganoderma*, *Hericium*, *Lentinula*, *Lentinus*, *Pleurotus*, *Tremella*, and *Volvariella* [10,11]. The four predominant genera in global cultivation are *Lentinula* (shiitake and allied species), *Pleurotus* (oyster mushroom), *Auricularia* (wood ear mushroom), and *Agaricus* (button mushroom and related forms) [11,12].

Recently, the therapeutic and medicinal properties of edible mushrooms have been receiving increased attention, particularly their immune-enhancing, antioxidant, antidiabetic, antitumor, and antimicrobial properties [1,3,4]. Phenolic compounds are a diverse group of secondary metabolites found in edible mushrooms; they are known for their potent antioxidant properties and associated health benefits. These compounds, which include phenolic acids, flavonoids, tannins, and lignans, significantly contribute to the therapeutic potential of mushrooms [1,2,3,4,13]. Additionally, polysaccharides from edible mushrooms have been shown to have diverse bioactive properties, including antidiabetic, antioxidant, antiviral, antilipidemic, antitumor, and immunomodulating properties [14,15]. Furthermore, ergothioneine, a naturally occurring sulfur-containing amino acid with powerful antioxidant properties, is abundant in edible mushrooms [16,17]. Beyond its role as an antioxidant, ergothioneine exhibits immunomodulatory, anti-inflammatory, cytoprotective, and neuroprotective activities, suggesting that it could have medical applications including managing conditions such as neurodegenerative diseases, cardiovascular disorders, and even cancer [17,18,19]. As researchers explored their biological significance, the bioactive compounds in mushrooms have emerged as valuable dietary components with both preventive and therapeutic health benefits. However, the centesimal content and bioactive compounds in edible mushrooms vary significantly depending on the species and strain, cultivation substrate, cultivation conditions, and stage of development. Mushrooms are commonly classified into young, middle, and mature stages based on consumer perception and prevailing market practices. Therefore, understanding the biochemical profile, including the centesimal composition and bioactive compounds, during different stages of mushroom development is essential for optimizing the harvest time and maximizing the health benefits.

Oyster mushrooms (*Pleurotus* spp.) rank among the most extensively cultivated and consumed edible mushrooms globally due to their high nutritional value, culinary adaptability, and straightforward cultivation process [11,12,20]. Beyond providing substantial amounts of protein, dietary fiber, vitamins, and essential minerals, oyster mushrooms possess numerous bioactive substances such as polysaccharides, phenolic compounds, and ergothioneine that enhance their beneficial health effects [21,22]. Due to their fast growth, adaptability to various substrates, and low production cost, oyster mushrooms also play a significant role in sustainable agriculture and food security, particularly in Southeast Asian countries, including Thailand. Several species of oyster mushrooms are widely consumed and commonly cultivated for commercialization by local farmers and entrepreneurs in Thailand for their economic potential [23]. They are frequently used in both home cooking and the food industry to make a variety of Thai cuisines. In Thai markets, oyster mushrooms are often sold at different stages of maturity, but many consumers believe that the young fruiting bodies are superior in taste, texture, and nutritional quality compared to those at the mature stage. This perception influences harvesting practices and market preferences, often leading to early harvesting to meet consumer demand [23,24,25]. However, scientific studies comparing the nutritional and bioactive compound profiles of young and mature oyster mushrooms remain limited. In northern Thailand, five species of oyster mushrooms, namely *P. citrinopileatus* (yellow oyster mushroom), *P. cornucopiae* (branched oyster mushroom), *P. djamor* (pink oyster mushroom), *P. ostreatus* (oyster mushroom), and *P. pulmonarius* (phoenix oyster mushroom), are commercially important and widely consumed. Therefore, these species were selected for this study. Additionally, three maturation stages (young, middle, and mature) were selected to evaluate how developmental stage influences the nutritional and bioactive compound compositions, which are critical for both consumption quality and potential functional applications. The aim of this study was to investigate the centesimal composition, bioactive compound content (including polysaccharides, ergothioneine, and phenolic compounds), antioxidant properties, and α-glucosidase inhibitory activity of these five edible oyster mushroom species cultivated in northern Thailand at three developmental stages.

## 2. Materials and Methods

### 2.1. Mushroom Strains

Five pure cultures of mushroom strains, *P. citrinopileatus* SDBR-CMUNK0954, *P. cornucopiae* SDBR-CMUNK1315, *P. djamor* SDBR-CMUNK1322, *P. ostreatus* SDBR-CMUNK1018, and *P. pulmonarius* SDBR-CMUNK1342, were used in this study. All the strains were obtained from the Culture Collection for Sustainable Development of Biological Resources, Faculty of Science, Chiang Mai University, Thailand. Each strain was cultivated on potato dextrose agar (PDA; Conda^®^, Madrid, Spain) at 30 °C in the dark.

### 2.2. Inoculum and Substrate Preparation

Sorghum grain (*Sorghum bicolor*) inoculum was prepared following the method described by Kumla et al. [26]. Sorghum grain was boiled for 10–15 min, after which, the excess water was drained off. Glass bottles (350 mL) were filled with 200 g of the boiled grain, sealed with cotton wool plugs, and sterilized by autoclaving at 121 °C for 30 min. After cooling, three mycelial plugs (5 mm in diameter) were taken from the edge of an actively growing colony and inoculated into the glass bottles, which were then incubated in darkness at 30 °C. Two-week-old sorghum grain completely covered by fungal mycelia was used as the inoculum.

Sawdust from rubber trees was used as the main substrate for cultivation. The sawdust was prepared following the method described by Jinanukul et al. [27] and supplemented with 5% rice bran, 1% CaCO_3_, 0.05% KH_2_PO_4_, and 0.15% MgSO_4_ based on dry weight. The humidity of the mixed substrate was adjusted to 60% to 70% using water. Eight hundred grams of mixed substrates were added to polypropylene bags (16.50 cm wide and 31.75 cm long), which were then closed with cotton-filled polyvinyl chloride pipe rings and wrapped with paper. After sterilization at 121 °C for 60 min, the bags were allowed to cool for 24 h and then used in the experiments.

### 2.3. Cultivation for Fruiting Body Production

Bag cultivation was employed in this study. Five grams of grain inoculum were inoculated into sterilized bags containing the cultivation substrate. The inoculated bags were incubated at room temperature (30 ± 2 °C) in the dark. A total of 40 bags were used for each strain. After one month, the fungal mycelia covered the substrate. The bags were transferred to a mushroom house at Farm Ban Hed Suphawat, a local facility located in Chiang Mai Province, Thailand, for fruiting body formation. The cotton-filled plug was removed. The experiment was conducted from June to September 2024. The conditions inside the mushroom house included temperatures ranging from approximately 25.5 °C to 33.5 °C, relative humidity levels between 75% and 80%, and a light intensity of about 250–550 lux with a 12 h light/12 h dark photoperiod.

### 2.4. Fruiting Body Harvesting, State Selection, and Sample Preparation

**Figure 1:**
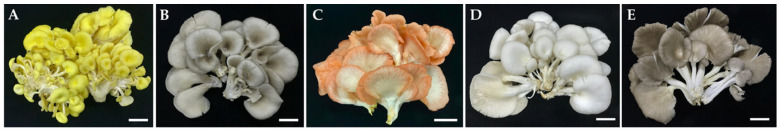
Fruiting bodies of five oyster mushroom species in this study: *Pleurotus citrinopileatus* (**A**), *P*. *cornucopiae* (**B**), *P. djamor* (**C**), *P. ostreatus* (**D**), and *P. pulmonarius* (**E**). Scale bars = 20 mm.

After one week in the mushroom house, the primordia of all the strains were observed and they developed into mature fruiting bodies within three days. Fruiting bodies were harvested from three successive crops during the cultivation period. After harvesting, the fruiting bodies were stored in ice-filled plastic boxes and transported to the laboratory within five hours for further analysis (Figure 1). The fruiting bodies were collected at three distinct stages: young, middle, and mature. The developmental stage for each oyster mushroom species was classified based on the morphological characteristics specific to the individual species (Table 1). The fruiting bodies were dried in a hot air oven at 45 °C until they were completely dry [28]. The moisture content of each sample was calculated based on wet and dry weight measurements and expressed as a percentage [29]. Each dried sample was ground using a Waring blender (New Hartford, CT, USA) and subsequently stored in a desiccator at room temperature (25 °C).

### 2.5. Determination of Centesimal Composition and Polysaccharide Content

The centesimal composition (including the ash, carbohydrate, fat, fiber, and protein contents) of each dried sample was determined using a method developed by the Association of Official Analytical Chemists (AOAC) [30] at the Central Laboratory Company Limited (Chiang Mai, Thailand). Five replicates were tested for each sample.

The polysaccharides in each sample were extracted using a hot water extraction method [31]. One gram of the ground mushroom sample was extracted with 10 mL of deionized water at 100 °C for 3 h in a water bath. After extraction, the sample was centrifuged at 25 °C at 4500× *g* for 5 min, and the supernatant was collected. The supernatant was then mixed with absolute ethanol at a 1:10 (*v*/*v*) ratio to precipitate the polysaccharides and then kept at 4 °C. After 24 h, the crude polysaccharides were collected by centrifugation at 25 °C at 4500× *g* for 5 min and subsequently freeze-dried until completely dry. The obtained crude polysaccharide was weighed and expressed as milligrams per gram of dry weight (mg/g dw). Five replicates were tested for each sample.

### 2.6. Determination of Ergothioneine Content

The ergothioneine content in the dried mushroom samples was measured according to the method of Zhu et al. [32] with some modifications. One gram of each ground mushroom sample was extracted with 10 mL of deionized water at 90 °C for 20 min in a water bath and then placed in an ultrasonic bath (Elma Transsonic Digital, Singen, Germany) set at 60 °C for 5 min. Each sample was centrifuged at 25 °C and 4500× *g* for 5 min. Each sample was filtered through a 0.22 μm nylon filter, and the ergothioneine content was analyzed using high-performance liquid chromatography (HPLC). The HPLC analysis was conducted using a Shimadzu Prominence UFLC system equipped with an LC-40D XS pump, SIL-40C XS autosampler, CTO-40C column oven, SCL-40 system controller, and SPD-M40 photodiode array detector (Shimadzu, Japan). Separation was achieved using a Cosmosil RP-18 column (250 × 4.6 mm, 5 μm) maintained at a constant temperature of 30 °C. The mobile phase consisted of 20% solvent A (10 mmol/L ammonium acetate in deionized water) and 80% solvent B (100% acetonitrile). The operating parameters were as follows: a flow rate of 1.0 mL/min, injection volume of 10 μL, and detection wavelength of 254 nm. Ergothioneine identification was verified through retention time matching and spiking samples with an ergothioneine reference standard. Quantitative analysis was performed by measuring the sample peak areas and comparing them with a standard ergothioneine calibration curve in the concentration range of 62.5–500 µg/mL (y = 0.0249x − 0.1509, R^2^ = 0.999). Five replicates were tested for each sample.

### 2.7. Determination of Total Phenolic Content and Phenolic Compound Profiles

The mushroom extract samples were prepared using absolute ethanol as the extraction solvent following method described by Kaewnarin et al. [33]. The total phenolic content and antioxidant and α-glucosidase inhibition potential of the extracts obtained from each mushroom species at each developmental state were determined. For the total phenolic content, the Folin–Ciocalteu assay was used following the method described by Thitilertdecha et al. [34]. The total phenolic content of the samples was quantified using a gallic acid calibration curve in the concentration range of 40–625 µg/mL (y = 0.0038x + 0.0433, R^2^ = 0.999). The results are expressed as milligrams of gallic acid equivalents per gram of dry weight (mg GAE/g dw). Five replicates of each sample extract were analyzed.

The phenolic compound profiles were quantified using HPLC (Shimadzu, Japan) using the instrumentation described above. Separation was achieved using a Mightysil RP-18 (250 × 4.6 mm, 5 μm) maintained at a constant temperature of 30 °C. The mobile phase consisted of solvent A (1% acetic acid in deionized water) and solvent B (100% acetonitrile). The following gradient program was used: 0–25 min, 0–8% B; 25–35 min, increase to 50% B; and 31–45 min, increase to 90% B. The flow rate was set to 0.5 mL/min, with an injection volume of 5 μL, and detection was carried out at an absorption wavelength of 280 nm. The presence of each phenolic compound was identified by comparing both the retention times and absorption spectra with those of standards; these phenolic compounds were caffeic acid, catechin, 3,4-dihydroxybenzoic acid, epicatechin, gallic acid, 4-hydroxybenzoic acid, quercetin, rutin, syringic acid, trans-cinnamic acid, trans-o-coumaric acid, trans-ferulic acid, vanillic acid, and vanillin (Supplementary Figure S1). Quantification was performed using calibration curves constructed for each individual standard using a concentration range of 62.5–1000 µg/mL (Supplementary Figure S2). Five replicates of each sample extract were analyzed.

### 2.8. Determination of Antioxidant Activity

In this study, the antioxidant activity of the ethanolic mushroom extracts was evaluated using three different assays: the 2,2′-azino-bis(3-ethylbenzothiazoline-6-sulfonic acid) (ABTS), 2,2-diphenyl-1-picrylhydrazyl (DPPH), and ferric reducing antioxidant power (FRAP) assays, which were carried out in accordance with the procedures described in Re et al. [35], Kaewnarin et al. [33], and Li et al. [36], respectively. Trolox was used as the reference standard. The ABTS, DPPH, and FRAP activities were determined using Trolox standard curves and expressed as Trolox equivalent antioxidant capacity per gram of dry weight (TE/g dw) (Supplementary Figure S3). ABTS activity was calculated using a standard concentration range of 10–150 µg/mL (y = −0.0035x + 0.6417, R^2^ = 0.999), while DPPH activity was calculated using a standard range of 2.5–25 µg/mL (y = −0.0282x + 0.872, R^2^ = 0.999). FRAP activity was calculated using a standard range of 10–200 µg/mL (y = 0.0061x + 0.0033, R^2^ = 0.999). Five replicates of each sample extract were analyzed.

### 2.9. Determination of α-Glucosidase Inhibitory Activity

The α-glucosidase inhibition potential of each ethanolic extract was assessed using the methodology described by Oki et al. [37] and Tanruean et al. [38]. Acarbose was used as the reference standard as it is a well-established synthetic α-glucosidase inhibitor. The results are expressed as the percentage of enzyme activity inhibited relative to the control. Five replicates of each sample extract were analyzed.

### 2.10. Statistical Analysis

Statistical analysis was conducted using SPSS 16.0 software. One-way ANOVA followed by Duncan’s Multiple Range Test were employed to identify significant differences between groups at the *p* < 0.05 significance level. Pearson correlation analysis was performed using SPSS software to assess the linear relationships between variables, with statistical significance set at *p* < 0.05.

## 3. Results and Discussion

### 3.1. Moisture Content in Fruiting Bodies

**Figure 2:**
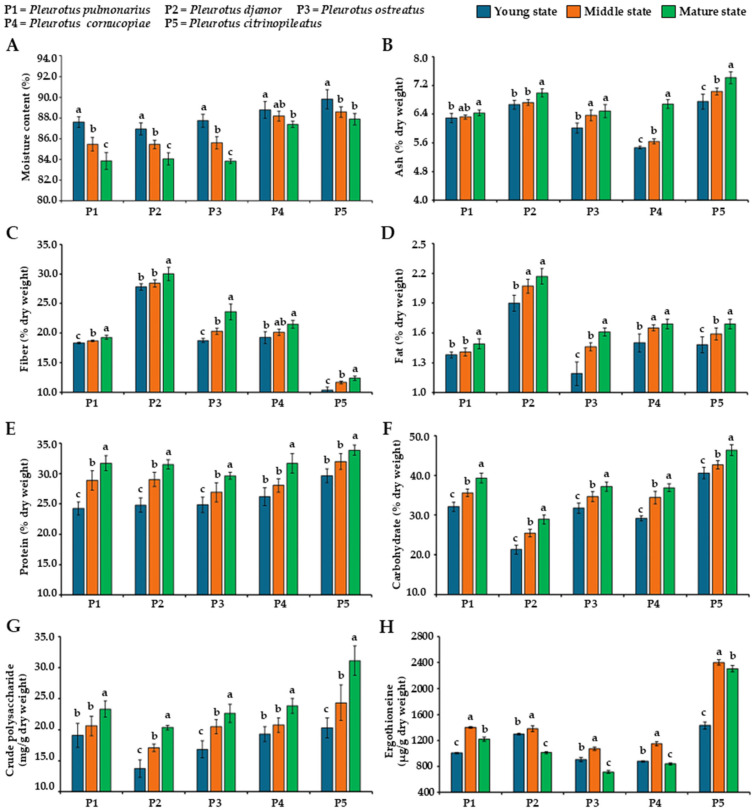
Moisture (**A**), ash (**B**), fiber (**C**), fat (**D**), protein (**E**), carbohydrate (**F**), crude polysaccharide (**G**), and ergothioneine (**H**) contents in fruiting bodies of five oyster mushroom species at different developmental stages. Results are presented as mean ± standard deviation. For each parameter, different letters above the bars indicate statistically significant differences (*p* < 0.05) between maturation stages within each mushroom species, as determined by Duncan’s Multiple Range Test.

The moisture content of fresh fruiting bodies of the five oyster mushroom species at various developmental stages is presented in Figure 2A. The results indicated that the moisture contents varied between mushroom species and developmental stages. The moisture content in the young, middle, and mature stages ranged from 86.9% to 89.8%, 85.43% to 88.57%, and 83.82% to 87.88%, respectively. The highest moisture content was observed in the young stage in all five oyster mushroom species, with a gradual decrease in water content as the mushrooms matured. Pearson correlation analysis (*p* < 0.05) revealed a significant negative correlation between developmental stage and moisture content across all five mushroom species, indicating that the moisture content decreased as the mushrooms matured (Table 2). Our findings showed that the moisture content of the five oyster mushrooms ranged from 83.82% to 89.8%, which aligns with previously reported values of 75% to 95% for various oyster mushroom species (e.g., *P*. *cystidiosus*, *P*. *djamor*, *P*. *eryngii*, *P. pulmonarius*, and *P. ostreatus*), depending on the species and stage when they are harvested [29,39,40,41,42,43,44]. The decrease in water content during fruiting body development from the young to mature stage observed in this study is consistent with the patterns reported in *Agaricus bisporus* [45] and *Lentinula edodes* [46], which also showed decreasing water contents as maturation progresses. These results are also supported by the findings of Herman and Bleichrodt [47] who found that more mature mushrooms have higher water contents, which is mostly due to water uptake during cell expansion.

### 3.2. Determination of Centesimal Composition

The centesimal composition, including the ash, fiber, fat, protein, and carbohydrate contents, of the various oyster mushroom species at the different developmental stages is presented in Figure 2B–F. The ash (5.47 to 7.41% dw), fiber (10.53 to 30.02% dw), fat (1.19 to 2.17% dw), protein (24.26 to 33.58% dw), and carbohydrate (21.28 to 46.31% dw) contents obtained in this study were within the previously reported ranges for edible wild mushrooms, particularly *Pleurotus* species, which varied from 4.1 to 13.6% dw, 0.39 to 44% dw, 0.4 to 9.5% dw, 11.0 to 45.7% dw, and 22.2 to 65.1% dw, respectively [3,7,29,44]. Among the species studied, *P. citrinopileatus* exhibited the highest levels of ash and carbohydrates. *Pleurotus djamor* showed the highest fiber and fat contents, whereas *P. citrinopileatus* recorded the lowest fiber content. The results indicate that the centesimal composition varied among the mushroom species and at different maturity stages. As maturation progressed, the ash, fiber, fat, protein, and carbohydrate contents increased in the five *Pleurotus* species. Pearson correlation analysis showed a positive correlation between developmental stage and centesimal content in all five mushroom species (Table 2), indicating that as development progressed, the centesimal content increased.

The results obtained in this study are consistent with those of Rahman et al. [48] who reported that the ash, fat, protein, and carbohydrate contents in *P. cystidiosus* increased during maturation, with the protein and carbohydrate levels decreasing after the mature stage. The observed increase in ash, fiber, fat, protein, and carbohydrate contents from the young to mature stages in mushrooms reflects the dynamic physiological and biochemical changes during development. As mushrooms mature, they accumulate minerals, chitin, glucans, and other structural polysaccharides in their cell walls, synthesize lipids in the cell membrane, and enhance the production of enzymes and structural proteins, resulting in an increase in the centesimal content due to these components [41,49,50]. However, Dikeman et al. [51] found that the ash and protein levels in *A. bisporus* slightly decreased from 12.5% dw to 11.9% dw and 38.1% to 37.5% dw, respectively, from the immature to mature stage. Several previous studies have reported that the centesimal content of mushrooms is significantly influenced by various factors, including the mushroom species, type of cultivation substrate, and maturity stage [52,53,54]. However, there is currently no standardized reference for clearly distinguishing between developmental stages for each mushroom species, and reports on the relationship between maturity stage and centesimal content remain limited. Therefore, future studies should be conducted on other mushroom species to establish standardized criteria for identifying developmental stages, which would support more accurate comparisons of centesimal compositions and improve consistency in mushroom research.

### 3.3. Determination of Polysaccharide Content

The crude polysaccharide content in the five oyster mushroom species was measured, revealing variations both between species and the different stages of maturity. The crude polysaccharide content increased as maturation progressed (Figure 2G). This result is supported by previous studies that reported that the crude polysaccharide content increases in mature mushrooms due to the accumulation of structural polysaccharides (chitin and glucans) in their cell walls [55,56]. An increase in the crude polysaccharide content is associated with an increase in the carbohydrate content during the development of mushrooms. In this study, Pearson correlation analysis revealed a significant positive correlation (*p* < 0.05) between developmental stage and polysaccharide content in all five mushroom species, indicating that the polysaccharide content increased with advancing developmental stage (Table 2). The polysaccharide contents (27.47 to 62.24 mg/g dw) obtained in this study were within the previously reported ranges for edible mushrooms, particularly *Pleurotus* species (*P. citrinopileatus*, *P. djamor, P. eryngii*, *P. pulmonarius*, and *P. ostreatus*), which varies from 18 to 236 mg/g dw [57,58,59,60,61,62,63,64]. Additionally, several previous studies have reported that the choice of extraction method influences the polysaccharide yield, with hot water extraction generally resulting in low or moderate yields compared to ultrasonic-assisted extraction, microwave-assisted extraction, enzyme-assisted extraction, and subcritical liquid extraction [14,59,60,63]. To achieve a more precise and comprehensive understanding of the changes in the polysaccharide content during maturation, future research should optimize the extraction and purification processes and perform monosaccharide composition and *β*-glucan analyses, structural characterization, and biological activity assessments, which would provide a deeper understanding of these polysaccharides’ properties and their potential applications.

### 3.4. Determination of Ergothioneine Content

**Figure 3:**
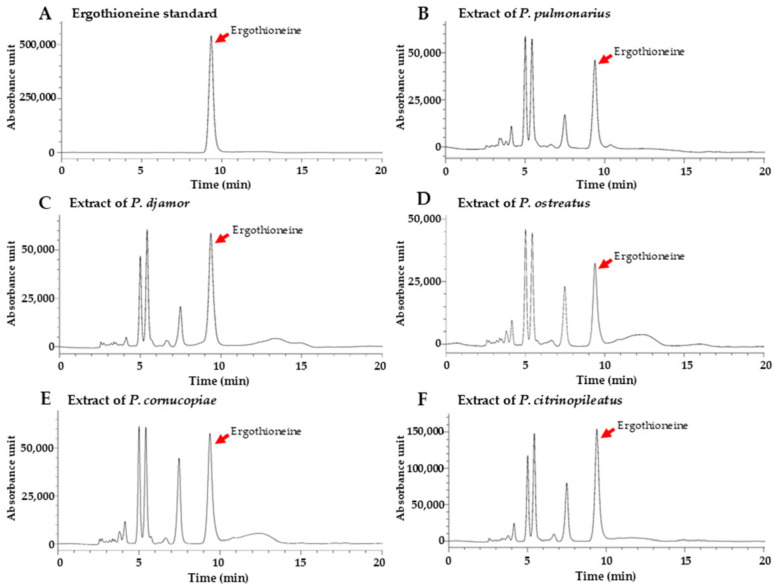
High-performance liquid chromatogram showing identification of ergothioneine in extracts from five oyster mushroom species at middle developmental stage. Ergothioneine standard (**A**), and *P. pulmonarius* (**B**), *P. djamor* (**C**), *P. ostreatus* (**D**), *P. cornucopiae* (**E**), and *P. citrinopileatus* (**F**) extracts.

Ergothioneine is a naturally occurring amino acid derivative with potent antioxidant activity. It is found in edible mushrooms, including *A. bisporus*, *Agrocybe cylindracea*, *Auricularia auricula*, *Flammulina velutipes*, *Ganoderma lucidum*, *Grifola frondosa*, *Lentinula edodes*, *Hericium erinaceus*, *Hypsizygus marmoreus*, *Pleurotus* species, and *Pholiota nameko*, with levels varying between mushroom species [17,18,65,66,67]. This compound possesses not only antioxidant activity, but also anti-inflammatory, immunomodulatory, neuroprotective, and cardioprotective effects [16,18]. HPLC analysis confirmed the presence of ergothioneine in the mushroom extracts based on an ergothioneine standard with a retention time of 9.36 min (Figure 3). Moreover, the identification of ergothioneine was confirmed by co-injection of the standard. Ergothioneine was found in *P. pulmonarius* (761.03 to 1253.52 µg/g dw), *P. djamor* (768.58 to 1226.73 µg/g dw), *P. ostreatus* (395.28 to 845.34 µg/g dw), *P. cornucopiae* (550.72 to 939.41 µg/g dw), and *P. citrinopileatus* (1290.15 to 2380.33 µg/g dw), with the levels varying between developmental stage and species. The highest ergothioneine content was found in *P. citrinopileatus*. Previous studies have reported the ergothioneine content in various *Pleurotus* species, including *P. albidus* (298 to 520 mg/g dw) [68], *P. citrinopileatus* (2850 to 11,800 mg/g dw) [66,69], *P. cystidiosus* (258 mg/g dw) [66], *P. eryngii* (234 to 1980 mg/g dw) [66,67,69,70], *P. ferulae* (464 mg/g dw) [66], *P. ostreatus* (994 to 9200 mg/g dw) [42,66,69,70], *P. pulmonarius* (275 to 638 mg/g dw) [68], and *P. salmoneostramineus* (1245 mg/g dw) [66]. Additionally, an ergothioneine content of 0.06 mg/g dw was reported in the waste from *P. cornucopiae* cultivation [67]. This study is the first to report the ergothioneine content in the fruiting bodies of *P. cornucopiae* and *P. djamor*.

In all five *Pleurotus* species, the ergothioneine content showed a developmental trend, increasing from the young to the middle stage, when the highest levels were observed, and then significantly decreasing in the mature stage (Figure 2H). According to the Pearson correlation analysis, a positive correlation was observed between developmental stage and centesimal content in all five mushroom species. This correlation was strong and statistically significant (*p* < 0.05) in *P. djamor* and *P. citrinopileatus* (Table 2). Previous studies have found that the cultivation substrate influences the ergothioneine content in mushrooms [68,69,71]. Therefore, identifying the mushroom species, developmental stage, and cultivation substrate that produce the highest ergothioneine levels could inform selective cultivation and harvesting practices aimed at enhancing the functional value of mushroom-based products. Moreover, such findings could support the use of ergothioneine-rich mushrooms as natural supplements or ingredients in health-promoting foods, offering a fungal-based alternative to synthetic antioxidants for the nutraceutical and pharmaceutical industries.

### 3.5. Determination of Total Phenolic Content and Phenolic Compound Profiles

**Figure 4:**
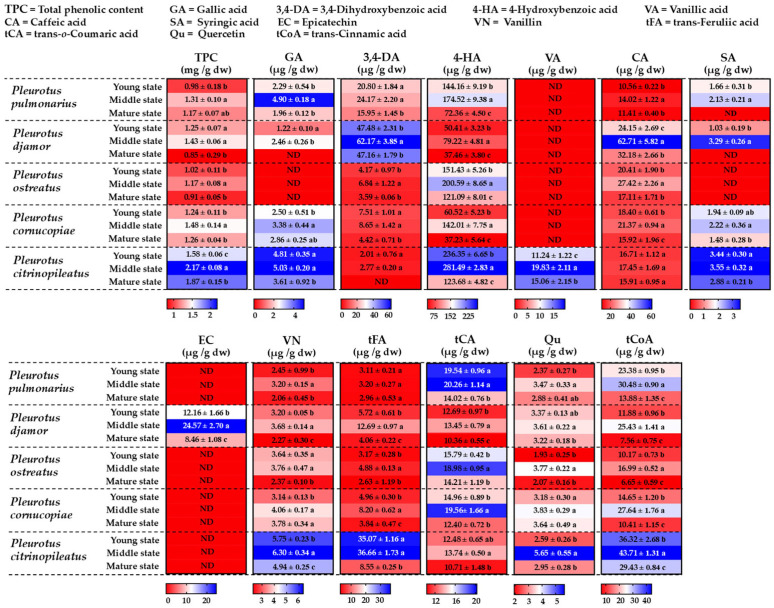
Heatmap showing total phenolic content and phenolic compound levels in extracts from five oyster mushroom species at different developmental stages. Data are presented as mean ± standard deviation. “ND” = not detected. Different letters indicate statistically significant differences (*p* < 0.05) between maturation stages within each mushroom species, as determined by Duncan’s Multiple Range Test.

The total phenolic content and antioxidant activity of the ethanolic extracts of the five *Pleurotus* species at different stages were analyzed; the results are shown in Figure 4. The total phenolic content was found to range from 0.82 to 2.17 mg GAE/g dw and varied between mushroom species and developmental stages (Figure 4). In all the mushroom species, the total phenolic content increased from the young to the middle stage and then decreased at the mature stage. The highest total phenolic content was found in the extract of *P. citrinopileatus*. The results of this study are consistent with those of previous studies showing that the phenolic content in edible mushrooms varies depending on the mushroom species [72,73]. The Pearson correlation analysis indicated a weak-to-moderate, non-significant, positive relationship between total phenolic content and developmental stage in all the mushroom species. The total phenolic contents obtained in this study fall within the previously reported range for edible mushrooms, including *Pleurotus* species (0.39 to 47.00 mg GAE/g dw), which is influenced by the mushroom species and extraction process [73,74,75,76,77,78,79,80]. Chilanti et al. [74] found that the total phenolic content in the fruiting body stage of *Pleurotus* species was higher than in the mycelial stage.

HPLC profiling of the ethanolic extracts revealed distinct phenolic compound profiles between the five *Pleurotus* species and the different developmental stages. The retention times of gallic acid, 3,4-dihydroxybenzoic acid, 4-hydroxybenzoic acid, catechin, vanillic acid, caffeic acid, syringic acid, epicatechin, vanillin, rutin, trans-feruliic acid, trans-o-coumaric acid, quercetin, and trans-cinnamic acid were 5.63, 10.92, 17.16, 19.20, 22.81, 24.57, 27.25, 30.63, 31.37, 32.34, 33.05, 34.54, 35.92, and 36.94 min, respectively (Supplementary Figure S2). The results indicate that 3,4-dihydroxybenzoic acid, 4-hydroxybenzoic acid, caffeic acid, vanillin, trans-ferulic acid, trans-o-coumaric acid, quercetin, and trans-cinnamic acid were detected in all *Pleurotus* species. Syringic acid was present in all species, except *P. ostreatus*. Additionally, vanillic acid and epicatechin were only found in *P. citrinopileatus* and *P. ostreatus*, respectively. The most abundant phenolic compounds across all the species were 4-hydroxybenzoic acid, trans-cinnamic acid, and trans-o-coumaric acid. The amount of each phenolic compound varied depending on the species and developmental stage. The levels of phenolic compounds increased from the young to the middle developmental stage, followed by a decrease at the mature stage. The highest levels of phenolic compounds were observed at the middle stage. The phenolic compounds found in this study were similar to those found in *Pleurotus* species in previous studies: caffeic acid, catechin, chlorogenic acid, cinnamic acid, coumaric acid, 3,4-dihydroxybenzoic acid, ferulic acid, 4-hydroxibenzoic acid, gallic acid, quercetin, syringic acid, vanillic acid, and vanillin [41,79,81,82,83,84]. In addition, the presence and levels of phenolic compounds in mushrooms were found to be influenced by the type of cultivation substrate, species, strain, as well as the extraction solvents and methods used [33,41,85].

### 3.6. Determination of Antioxidant Activity

**Figure 5:**
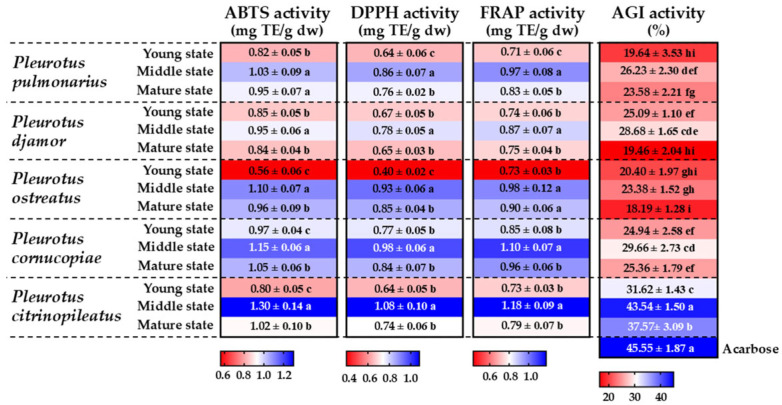
Heatmap showing ABTS, DPPH, FRAP, and α-glucosidase inhibitory (AGI) activities of extracts from five oyster mushroom species at different developmental stages. Data are presented as mean ± standard deviation. For ABTS, DPPH, and FRAP activities, different letters indicate statistically significant differences (*p* < 0.05) between maturation stages within each mushroom species, as determined by Duncan’s Multiple Range Test. For α-glucosidase inhibitory activity, different letters indicate statistically significant differences (*p* < 0.05) between mushroom species and acarbose, as determined by Duncan’s Multiple Range Test.

The antioxidant activity of the ethanolic extracts of the various *Pleurotus* species was evaluated using three different methods: the ABTS, DPPH, and FRAP assays. The antioxidant activities in the ABTS and DPPH assays are assessed by measuring the reduction in absorbance of the ABTS and DPPH radicals, which indicates the degree of scavenging by antioxidants [86,87]. On the other hand, the FRAP assay evaluates the antioxidant potential by measuring the reduction in ferric (Fe^3+^) to ferrous (Fe^2+^) ions [87]. The ABTS, DPPH, and FRAP results are displayed in Figure 5. All the extracts displayed antioxidant activity, with the ABTS, DPPH, and FRAP activities ranging from 0.56 to 1.03, 0.40 to 1.08, and 0.43 to 1.18 mg TE/g dw, respectively. These results are supported by previous studies that found variations in antioxidant activity among different *Pleurotus* species [29,70,74,75,79,82]. However, the use of different units of measurement in previous studies [such as inhibition percentage, half-maximal inhibitory concentration (IC_50_), or comparisons with various positive standards] makes it difficult to compare their results with those of the present study. Our results revealed that the antioxidant activity increased from the young to the middle stage, reaching its highest point, and then declined significantly in the mature stage. Among the samples, the extract of middle-stage *P. citrinopileatus* demonstrated the highest ABTS, DPPH, and FRAP activities, while the extract of young-stage *P. ostreatus* showed the lowest. The results of this study are consistent with those of previous findings showing that closed fruiting bodies (young and middle stages) of *A. bisporus* exhibit higher antioxidant activity compared to opened fruiting bodies (mature stage) [88]. González-Palma et al. [76] reported that the ABTS, DPPH, and FRAP antioxidant activities of *P. ostreatus* extracts varied between developmental stages, with the fruiting stage displaying higher antioxidant activity than the mycelial stage. Raman et al. [89] found that the antioxidant activities measured by the DPPH and lipid peroxidation assays were higher in the fruiting body stage of *P. djamor* var. *roseus* compared to both the mycelial and primordial stages. In this study, Pearson correlation analysis indicated a non-significant positive relationship between increasing developmental stage and antioxidant activity in all five mushroom species, except for the DPPH activity of *P*. *ostreatus*, which showed a significant positive correlation (Table 2). Furthermore, the total phenolic compound content and ABTS, DPPH, and FRAP activities showed a moderate positive correlation (Table 3). A significant positive correlation (*p* < 0.05) was observed between total phenolic content and both ABTS (*r* = 0.594) and FRAP (*r* = 0.589) activities in the mushroom extracts, suggesting that phenolic compounds play a key role in their antioxidant activity. This finding is consistent with those of previous studies that report that phenolic compounds are major contributors to the antioxidant activity in edible mushrooms. For instance, a study by Bristy et al. [90] found a significant correlation between total phenolic content and antioxidant activity in *Calocybe indica*, *Ganoderma lucidum*, and *G. tropicum*. Similarly, Kim et al. [91] reported a positive correlation between total phenolic content and antioxidant capacity in ten edible mushroom species, including *P. eryngii* and *P. ostreatus*. Moreover, Dubost et al. [92] reported a positive correlation between total phenolic content and antioxidant capacity in *A. bisporus*, *G. frondosa*, *L. edodes*, *P. eryngii*, and *P. ostreatus*. Therefore, this strong association highlights the importance of phenolic accumulation during maturation, suggesting that the harvesting stage could influence the functional properties of these mushrooms.

### 3.7. Determination of α-Glucosidase Inhibitory Activity

Inhibiting the α-glucosidase enzyme can help lower and manage high blood sugar levels as this enzyme plays a key role in the breakdown of carbohydrates into glucose, leading to increased blood sugar levels [93,94,95]. This study determined the α-glucosidase inhibitory activity of the extracts of the five *Pleurotus* species by measuring their percentage of inhibition. These results were compared to those of acarbose, a commonly used antidiabetic drug. The findings showed that all the extracts demonstrated *α*-glucosidase inhibitory effects, although the inhibition percentages differed between the samples (Figure 5). Acarbose, the reference drug, exhibited 45.55% α-glucosidase inhibitory activity while the activities of the extract samples ranged from 18.19% to 43.54%. Among all five *Pleurotus* species, the fruiting bodies at the middle developmental stage exhibited the highest α-glucosidase inhibitory activity. The extracts of *P. citrinopileatus* exhibited higher α-glucosidase inhibitory activity compared to those of the other species. The α-glucosidase inhibitory activities of the mushroom extracts were lower than that of acarbose, the standard reference. However, the extract of *P. citrinopileatus* at the middle developmental stage showed no significant difference from acarbose, indicating comparable inhibitory efficacy. These findings are consistent with previous studies demonstrating that extracts of *P. citrinopileatus* and *P. ostreatus* possess α-glucosidase inhibitory activities ranging from about 5% to 57.14%, with variations between different species and extraction solvents [79,95]. Wahab et al. [96] found that protein fractions extracted from *P. pulmonarius* fruiting bodies exhibited α-glucosidase inhibitory effects. To our knowledge, this study represents the first report on the α-glucosidase inhibitory activities of *P. cornucopiae* and *P. djamor*. In vitro assays from previous studies showed that mushroom phenolic compounds can also inhibit the activity of α-glucosidase enzymes [33,97,98]. In this study, Pearson correlation analysis revealed a strong, significant, positive correlation between total phenolic content and α-glucosidase inhibitory activity, indicating that a higher total phenolic content is associated with increased α-glucosidase inhibitory activity (Table 3). These results are consistent with those of Kumla et al. [28] who found a strong significant correlation between the total phenolic content of extracts from edible *Amanita* mushrooms and α-glucosidase inhibitory activity. Similarly, Kaewnarin et al. [33] reported that mushroom species with higher polyphenol contents exhibit greater α-glucosidase inhibitory activities. Other investigations also found that natural substances with higher phenolic contents exhibit stronger α-glucosidase inhibitory effects [98,99,100].

## 4. Conclusions

This study investigated the nutritional content, bioactive component contents (polysaccharides, ergothioneine, and phenolic compounds), antioxidant potential, and α-glucosidase inhibitory effects of five cultivated edible oyster mushroom species (*P. citrinopileatus*, *P. cornucopiae*, *P. djamor*, *P. ostreatus*, and *P. pulmonarius*) from northern Thailand at three maturation stages. The findings revealed that the centesimal composition and polysaccharide content increased with maturation in all species. Notably, the middle developmental stage consistently exhibited the highest levels of ergothioneine, total phenolics, and individual phenolic compounds in all five species. Twelve phenolic compounds were identified, with 4-hydroxybenzoic acid, trans-cinnamic acid, and trans-o-coumaric acid being the most abundant. All mushroom extracts demonstrated robust antioxidant activity (ABTS, DPPH, and FRAP activities) and α-glucosidase inhibitory activity, with both bioactivities reaching their maximum levels at the middle maturation stage. Importantly, this study provides the first report on the ergothioneine content and α-glucosidase inhibitory activity in *P. cornucopiae* and *P. djamor*. Consequently, the findings of this study enhance our understanding of how developmental stage affects both the nutritional and functional properties of commercially important *Pleurotus* species. From an agricultural perspective, growers can optimize harvest timing to maximize the nutritional value and bioactive compound content depending on the intended application, whether for fresh consumption or functional food production (e.g., supplements, food additives, or extracts), and based on consumer and food developer demands, thereby enhancing the practical utility of oyster mushroom cultivation in Thailand and beyond. Future research should focus on conducting in vivo experiments to evaluate the bioavailability and efficacy of the antioxidant and α-glucosidase inhibitory activities, as well as investigating a broader range of bioactivities (e.g., anti-inflammatory, antimicrobial, and glycemic control effects) through both in vitro and in vivo approaches.

## Figures and Tables

**Table 1:** Classification of developmental stages in oyster mushroom species based on morphological characteristics.

*Pleurotus* Species	Morphological Characteristics		
	Young Stage	Middle Stage	Mature Stage
*P. citrinopileatus*	Cap: 5–20 mm in diameter, bright yellow, convex with curled edges. Stipe: 10–20 mm long.	Cap: 21–40 mm in diameter, yellow, expanded and beginning to flatten. Stipe: elongated, 21–40 mm long.	Cap: ≥41 mm in diameter, yellow to pale yellow, flat with slightly downturned edges. Stipe: ≥41 mm long, cylindrical, and slender.
*P. cornucopiae*	Cap: 10–25 mm in diameter, gray, convex with inrolled margins. Stipe: 20–30 mm long	Cap: 26–50 mm in diameter, gray to light brownish, flattened with margins starting to expand. Stipe: elongated, 31–40 mm long.	Cap: ≥51 mm in diameter, pale brown, fully expanded or slightly depressed in the center. Stipe: ≥41 mm long, often curved or tapering.
*P. djamor*	Cap: 10–25 mm in diameter, bright pink with edges curled inward. Stipe: 5–10 mm long.	Cap: 26–50 mm in diameter, pink to light pink, flat with wavy edges. Stipe: 11–25 mm long.	Cap: ≥51 mm in diameter, pale pink to salmon pink, wide and flat with wavy edges that may curl upward. Stipe: ≥26 mm long.
*P. ostreatus*	Cap: 10–25 mm in diameter, gray to brown with tightly curved edges. Stipe: 5–15 mm long.	Cap: 26–50 mm in diameter, light gray with tightly curved edges. Stipe: 16–30 mm long.	Cap: ≥51 mm in diameter, pale gray to white, flat to funnel shaped. Stipe: ≥31 mm long, cylindrical, and slender.
*P. pulmonarius*	Cap: 10–30 mm in diameter, light gray, convex with curled edges. Stipe: 5–20 mm long.	Cap: 31–60 mm in diameter, light gray to pale gray, flattened. Stipe: 21–40 mm long.	Cap: ≥61 mm in diameter, pale gray to light brown, flat to funnel-shaped. Stipe: ≥41 mm long, cylindrical, and slender.

**Table 2:** Pearson correlation coefficients between increasing maturity stages and centesimal, polysaccharide, ergothioneine, and total phenolic contents, and antioxidant and α-glucosidase inhibitory activities of each oyster mushroom species.

Parameter	Pearson Correlation Coefficients with Increasing Maturity (*r*/*p*-Value)				
	*P. pulmonarius*	*P. djamor*	*P. ostreatus*	*P. cornucopiae*	*P. citrinopileatus*
Moisture	−0.922 */<0.01	−0.928 */<0.01	−0.960 */<0.01	−0.737 */0.02	−0.784 */<0.01
Ash	0.549 */0.03	0.787 */<0.01	0.783 */<0.01	0.941 */<0.01	0.872 */<0.01
Fiber	0.864 */<0.01	0.763 */<0.01	0.914 */<0.01	0.831 */<0.01	0.896 */<0.01
Fat	0.741 */0.02	0.833 */<0.01	0.921 */<0.01	0.793 */<0.01	0.833 */<0.01
Protein	0.924 */<0.01	0.934 */<0.01	0.871 */<0.01	0.862 */<0.01	0.909 */<0.01
Carbohydrate	0.948 */<0.01	0.955 */<0.01	0.893 */<0.01	0.932 */<0.01	0.889 */<0.01
Polysaccharide	0.761 */<0.01	0.858 */<0.01	0.887 */<0.01	0.853 */<0.01	0.896 */<0.01
Ergothioneine	0.542/0.06	0.733 */<0.01	0.522/0.08	0.113/0.72	0.83 */<0.01
TPC	0.455/0.14	0.587/0.55	0.347/0.27	0.573/0.86	0.466/0.12
ABTS Activity	0.499/0.98	0.121/0.71	0.398/0.72	0.370/0.23	0.389/0.21
DPPH Activity	0.496/0.10	0.08/0.78	0.780 */<0.01	0.283/0.37	0.185/0.56
FRAP Activity	0.397/0.20	0.267/0.93	0.467/0.40	0.368/0.24	0.114/0.723
AGI Activity	0.446/0.14	0.560/0.58	0.353/0.26	0.584/0.85	0.466/0.13

“*” indicates a significant correlation (*p* < 0.05) between each parameter and the increasing maturity stage of each mushroom species. TPC = total phenolic compound; AGI = α-glucosidase inhibitory.

**Table 3:** Pearson correlation coefficients (*r*) between total phenolic content and antioxidant and α-glucosidase inhibitory activities of oyster mushrooms.

Parameter	Pearson Correlation Coefficients (*r*/*p*-Value)			
	Antioxidant Activity			α-Glucosidase Inhibitory Activity
	ABTS Activity	DPPH Activity	FRAP Activity	
Total phenolic content	0.594 */0.02	0.493/0.06	0.589 */0.04	0.995 */<0.01

“*” indicates a significant correlation (*p* < 0.05) between total phenolic compound content and measured activity.

## Data Availability

The original contributions presented in this study are included in the article/Supplementary Materials. Further inquiries can be directed to the corresponding author.

## Supplementary Materials

The following supporting information can be downloaded at: https://www.mdpi.com/article/10.3390/foods14203511/s1, Supplementary Figures S1–S3. High-performance liquid chromatograms and standard curves for total phenolic content, antioxidant activity, and individual phenolic compounds. Figure S1. High performance liquid chromatogram showing the identification of phenolic compounds in extracts from five oyster mushroom species at the middle developmental stage. Phenolic compound standards (A), extract of *P. pulmonarius* (B), *P. djamor* (C), *P. ostreatus* (D), *P. cornucopiae* (E), and *P. citrinopileatus* (F). Figure S2. Standard curve for ergothioneine, total phenolic compound, and phenolic compounds. Figure S3. Trolox standard curves for ABTS, DPPH and FRAP activities.
