# Supplementary material for: Centesimal Composition, Bioactive Compounds, Antioxidant and α-Glucosidase Inhibitory Activities of Commercial Edible Oyster Mushrooms at Different Maturity Stages in Northern Thailand
Source: Foods. 2025 Oct 15;14(20):3511. doi: 10.3390/foods14203511 (PMC12564318; doi:10.3390/foods14203511)
Supplement: Supplementary file 1 [file foods-14-03511-s001.zip › foods-3908837-supplementary.pdf]

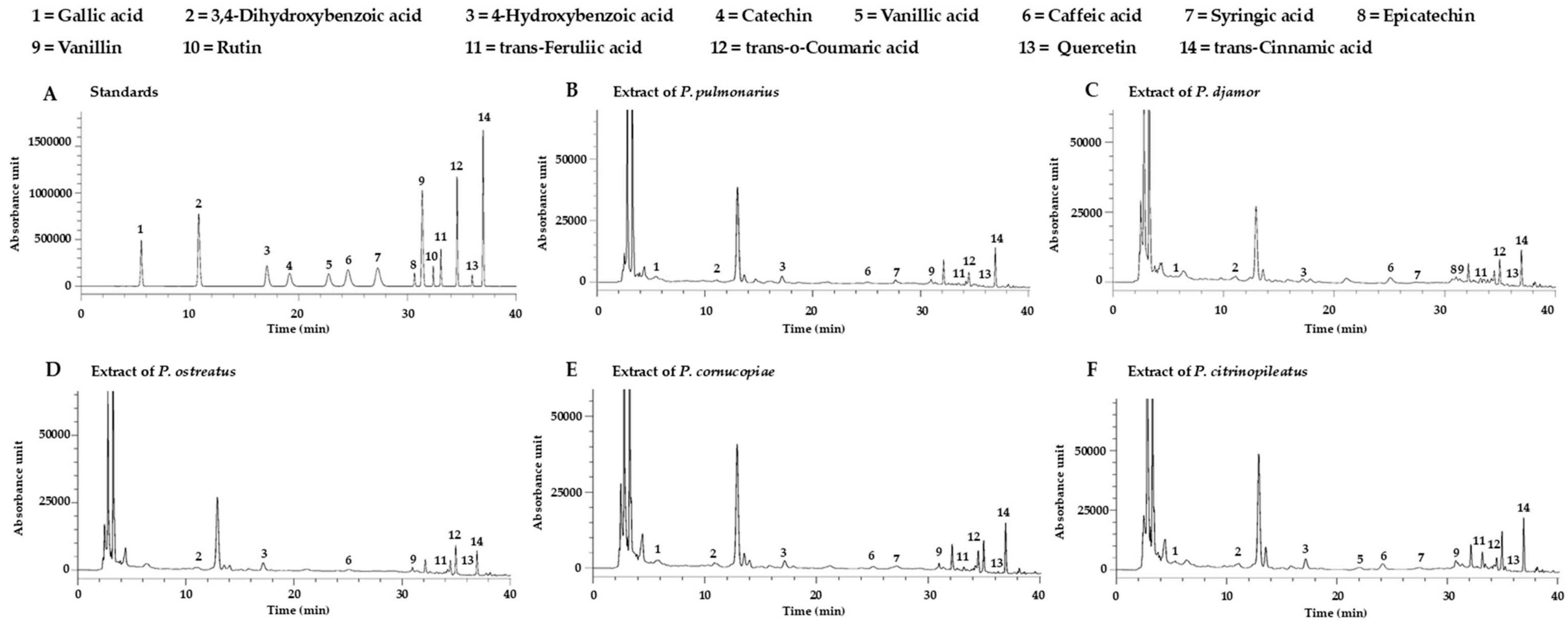

Figure S1. High performance liquid chromatogram showing the identification of phenolic compounds in extracts from five oyster mushroom species at the middle developmental stage. Phenolic compound standards (A), extract of *P. pulmonarius* (B), *P. djamor* (C), *P. ostreatus* (D), *P. cornucopiae* (E), and *P. citrinopileatus* (F).

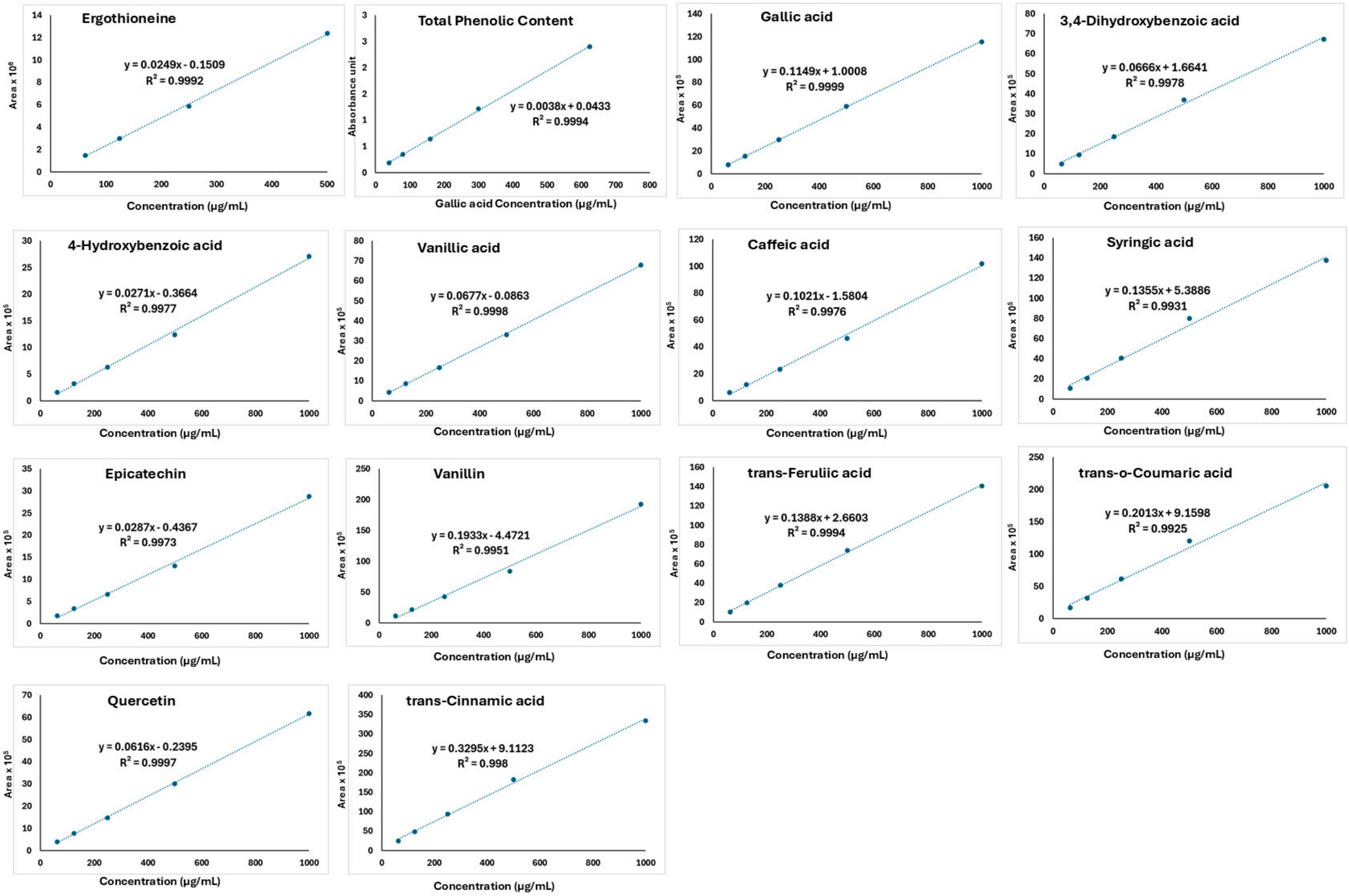

Figure S2. Standard curve for ergothioneine, total phenolic compound, and phenolic compounds.

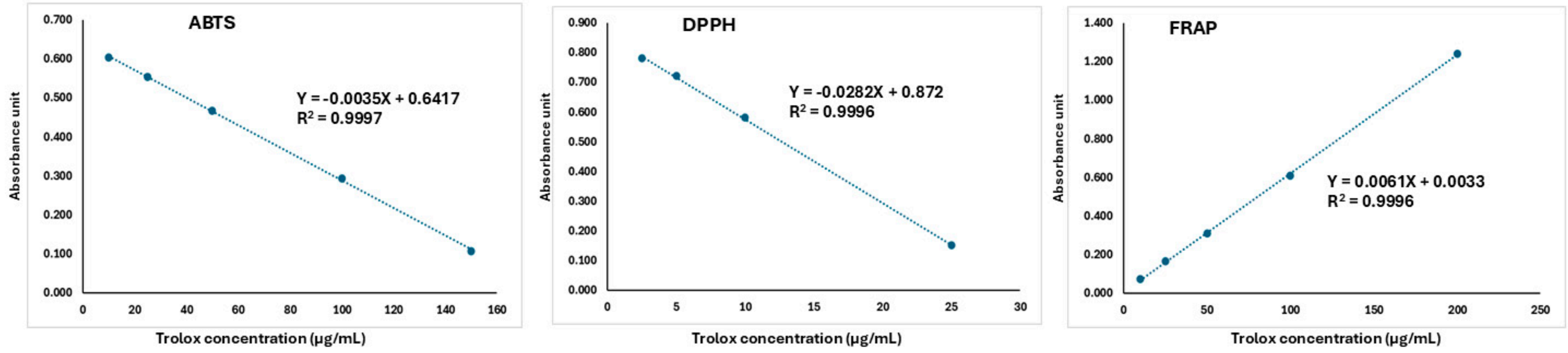

Figure S3. Trolox standard curves for ABTS, DPPH and FRAP activities.
